# Hypothyroxinemia Induced by Mild Iodine Deficiency Deregulats Thyroid Proteins during Gestation and Lactation in Dams

**DOI:** 10.3390/ijerph10083233

**Published:** 2013-08-02

**Authors:** Wei Wei, Yi Wang, Jing Dong, Yuan Wang, Hui Min, Binbin Song, Zhongyan Shan, Weiping Teng, Qi Xi, Jie Chen

**Affiliations:** 1 Department of Occupational and Environmental Health, School of Public Health, China Medical University, 92 North 2nd Road, Shenyang 110001, China; E-Mails: weiwei840403@163.com (W.W.); wangyi@mail.cmu.edu.cn (Y.W.); dj814@163.com (J.D.); wangyuan041320@126.com (Y.W.); minhui19890706@163.com (H.M.); mbin0263@163.com (B.S.); qxi@uthsc.edu (Q.X.); 2 Liaoning Provincial Key Laboratory of Endocrine Diseases, the First Hospital of China Medical University, Shenyang 110001, China; E-Mails: shanzhongyan@medmail.com.cn (Z.S.); twp@vip.163.com (W.T.); 3 Department of Physiology, the University of Tennessee Health Science Center, Memphis, TN 38163, USA

**Keywords:** mild iodine deficiency, hypothyroxinemia, gestation, lactation, thyroid

## Abstract

The main object of the present study was to explore the effect on thyroidal proteins following mild iodine deficiency (ID)-induced maternal hypothyroxinemia during pregnancy and lactation. In the present study, we established a maternal hypothyroxinemia model in female Wistar rats by using a mild ID diet. Maternal thyroid iodine content and thyroid weight were measured. Expressions of thyroid-associated proteins were analyzed. The results showed that the mild ID diet increased thyroid weight, decreased thyroid iodine content and increased expressions of thyroid transcription factor 1, paired box gene 8 and Na^+^/I^−^ symporter on gestational day (GD) 19 and postpartum days (PN) 21 in the maternal thyroid. Moreover, the up-regulated expressions of type 1 iodothyronine deiodinase (DIO1) and type 2 iodothyronine deiodinase (DIO2) were detected in the mild ID group on GD19 and PN21. Taken together, our data indicates that during pregnancy and lactation, a maternal mild ID could induce hypothyroxinemia and increase the thyroidal DIO1 and DIO2 levels.

## 1. Introduction

Iodine is an essential trace element for the thyroid gland to produce triiodothyronine (T_3_) and thyroxine (T_4_), which are the only iodine-containing hormones in vertebrates [1]. An adequate supply of iodine is important for the thyroid to exert its widespread effects [2]. Over 1.9 billion people around the World are affected by inadequate iodine nutrition [3]. Iodine deficiency (ID) can lead to hypothyroidism [4]. Maternal ID-induced clinical and subclinical hypothyroidism during pregnancy and lactation results in a decrease in IQ of the child [4,5,6].

Besides hypothyroidism, ID can also cause hypothyroxinemia, characterized by a condition of subnormal T_4_ concentrations in the blood, where circulating free thyroxine (FT_4_) is low, but with no change in free triiodothyronine (FT_3_) or thyroid stimulating hormone (TSH). Different from hypothyroidism, hypothyroxinemia does not necessarily result in clinical or even “subclinical” hypothyroidism. Globally, mild ID is recognized as the most common reason causing hypothyroxinemia [7,8]. Epidemiology studies show that the possibility of neurodevelopmental impairment of children due to early maternal hypothyroxinemia is greater than in adequately-treated congenital hypothyroidism [9,10]. During pregnant and lactation, more dietary iodine is necessary to maintain the normal state of the maternal thyroid. Moreover, the brain development of the fetus depends on maternal thyroid function [7,11]. However, relatively little is known about the effect on thyroidal proteins following mild ID-induced maternal hypothyroxinemia in gestational and lactational dams.

Thyroid transcription factor 1 (TTF1), alternatively known as Nkx2-1 (homeobox protein Nkx2-1), and paired box gene 8 (PAX8) play pivotal roles, both in the development of the thyroid and in maintaining normal thyroid function [12,13]. The transcription factors TTF1 and PAX8 are expressed in the developing thyroid to adulthood [14,15]. TTF1 and PAX8 have been shown to regulate expression of thyroid-specific genes, such as Na^+^/I^−^ symporter (NIS) [16,17]. As the thyroid iodide transporter, NIS plays a key role in iodide concentration that is an essential step in thyroid hormone (TH) synthesis [18]. Many kinds of thyroid disorders and ID are implicated in the alterations of the NIS gene [19,20]. Given that the TTF1, PAX8 and NIS are pivotal regulators of transporting iodine in the thyroid, we speculated that mild ID diet may induce mild ID by impacting the expressions of thyroidal TTF1, PAX8 and NIS. Moreover, type 1 iodothyronine deiodinase (DIO1) and type 2 iodothyronine deiodinase (DIO2) activate TH by catalyzing the outer ring deiodination of the T_4_ to bioactive T_3_, thereby they play a role in TH activation. Therefore, we also hypothesized that, in the gestational and lactational dams, mild ID may induce hypothyroxinemia and disturb thyroidal DIO1 and DIO2 expressions. To test the hypothesis, we tried to use a mild ID diet to establish a maternal hypothyroxinemia model in Wistar rats. In addition to the negative control group, a maternal hypothyroidism model was induced by a severe ID diet. Maternal mild ID was monitored by detecting thyroid weight, thyroid iodine content and the expression levels of thyroidal TTF1, PAX8 and NIS on gestational day (GD) 19 and postpartum days (PN) 21. Moreover, the expressions of DIO1 and DIO2 on GD19 and PN21 were detected.

## 2. Materials and Methods

### 2.1. Animals

Female Wistar rats (130–150 g) were obtained from the Center for Experimental Animals at China Medical University (Shenyang, China) with a National Animal Use License number of SCXK-LN2003-0009. Animal use has been approved by Animal Use and Care Committee at China Medical University. Rats were housed at temperature 24 ± 1 °C with a 12/12 h light/dark cycles. Food and water were provided *ad libitum*. Animals were kept for 1 week. The female Wistar rats were randomly assigned into three groups: control group, mild ID group and severe ID group. Every group received an ID diet (iodine content: 60 ± 1.5 ng/g) and drunk different iodine concentration deionized water supplemented with KI. The rats’ iodine intake of the whole day was estimated as follows: 7.0 μg/d, 3.0 μg/d and 1.5 μg/d. The female rats were fed the diet for three months. At the end of the three months, blood was obtained from from the jugular vein of the rats under slight ether anesthesia for subsequent measurement of TH and TSH by a supersensitive chemiluminescence immunoassay (IMMULITE; Diagnostic Products Corporation, Los Angeles, CA, USA). The female rats were then mated with normal male rats (♀:♂ = 2:1). The day of the vaginal plug was taken as GD0. Until PN21, the dams of control, mild ID and severe ID groups were fed with the former methods. On GD19 and PN21, five dams were randomly taken per group. After ether anesthesia, heart blood samples were obtained for TH and TSH analysis. The sensitivity of detection for FT_3_ was 1.5–61 pmol/L, FT_4_ was 3.9–77.2 pmol/L and TSH was 0.02–75 mIU/L.

### 2.2. Iodine Deficient Diet

The ID diet was obtained from the ID area. To prevent contamination with small amounts of T_4_ and T_3_, the ID diet we used does not contain any component of animal origin. It is nutritionally inadequate, even when supplemented with KI, which maybe lead to poor growth of dams that are fed this diet. To avoid this possible confounding factor in this study, each kilogram of the basic iodine-deficient diet (corn (30%), rice (30%), soybean (40%)) was fortified with 35 g of mineral mixture, and 10 g of the vitamin mixture described following the AIN-93G purified rodent diet guidelines [21], KI excluded, and with 13 g of l-lysine, 2.1 g of l-tryptophane, 4.6 g of l-methione, 6.7 g of l-threonine, and 1 g of l-choline [21]. In addition, 10 mL of corn oil was added to each kg of diet to ensure an adequate supply of essential fatty acids. With this supplemented ID diet dams grew normally when also supplemented with KI (7.0 μg/d).

### 2.3. Measurement of Thyroid Iodine Content

Thyroid glands were collected from five dams on GD19 and PN21 for measurement of thyroid iodine content by the ammonium persulfate-arsenic cerium catalytic spectrophotometry [22].

### 2.4. Western Blotting

On GD19 and PN21, rats in each group, were deeply anesthetized and euthanized by ether. The thyroid glands of the five selected dams per group at each measured day were rapidly dissected. A tissue sample of each rat was homogenized in 250 μL of buffered isotonic cocktail containing protease and phosphatase inhibitors. The sample was sonicated and incubated on ice for 30 min and then centrifuged at 13,000× g for 10 min at 4 °C. The resulting supernatant was re-centrifuged and saved. The protein was estimated by Pierce BCA Protein Assay Kit (Thermo Scientific, Waltham, MA, USA). Samples were stored at −70 °C until analyzed.

Tissue lysates of each rat sample were diluted to the protein concentration of 3 μg/μL and were boiled for 5 min. Ten μL aliquots of each sample (30 μg total protein) were loaded onto 10% SDS-acrylamide gels. Proteins were separated by application of a constant voltage of 100 V for 1.5 h and then transferred onto PVDF membranes at a constant voltage of 10 V for 45 min. After blocking the nonspecific sites with PBS containing 0.1% Tween 20 (PBST) and 5% defatted dried milk, membranes were washed and incubated with primary antibody (rabbit anti-TTF1, 1:200 dilution; rabbit anti-PAX8, 1:200 dilution; rabbit anti-NIS, 1:200 dilution; rabbit anti-DIO1, 1:200 dilution; rabbit anti-DIO2, 1:200 dilution; rabbit anti-β-actin, 1:2,000 dilution. All the antibodies were produced by Santa Cruz Biotechnology, Santa Cruz, CA, USA) for 2 h at room temperature, then incubated with horseradish peroxidase-conjugated secondary antibody (goat anti-rabbit, 1:3,000 dilution, Zhongshan Biotechnology, Beijing, China). Blots were developed with the Easy Enhanced Chemiluminescence Western Blot Kit (Transgen Biotech, Beijing, China). Initial control experiments determined the optimal time for exposing the blot to film, which was maintained throughout the experimental procedure. Membranes were exposed to film for the optimal time for each antibody and developed. Protein bands were subsequently quantified with an image analysis program (Gel mage System Version 4.00) and the data were recorded, with the net optical density corrected for background chemiluminescence. For each blot, the β-actin lanes were analyzed as a quality control. The signals from target bands on a gel were normalized to the average signal for the quality control sample bands to simplify comparison across gels and reduce inter-gel variability.

### 2.5. Statistics

All analyses were carried out using SPSS software, version 16.0 (SPSS Inc., Chicago, IL, USA). Data are presented as the mean ±SD. *p* < 0.05 was considered statistically significant. A one-way analysis of variance followed by Student-Newman-Keuls test was used to compare the treated groups with the control.

## 3. Results

### 3.1. Animal Model

On GD19 and PN21, the serum FT_4_ concentration in the mild ID and severe ID treatment groups was significantly lower than the controls (*p* < 0.05). Compared with the control groups, FT_3_ and TSH concentration in the mild ID group had no significant difference on GD19 and PN21. However, we did obtain the significant decrease in TH and increase in TSH on GD19 and PN21 (*p* < 0.05). Alterations in circulating levels of TH and TSH confirmed hypothyroxinemia in the mild ID dams and hypothyroidism in the severe ID dams [23].

### 3.2. Increased Thyroid Gland Weight and Decreased Thyroid Iodine Content

**Figure 1:**
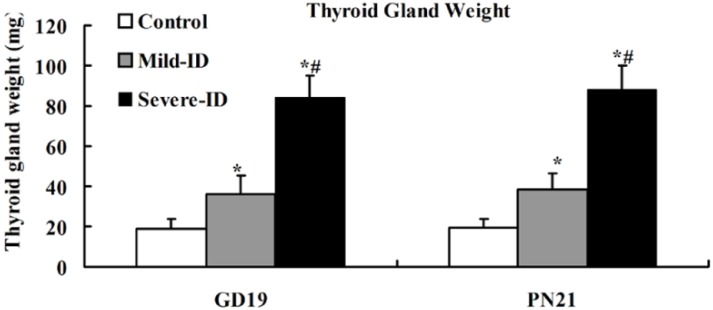
The increased thyroid weight in the thyroid in all groups on GD19 and PN21 (n = 5). * compared to the control, *p* < 0.05; # compared to the mild ID, *p* < 0.05.

**Figure 2:**
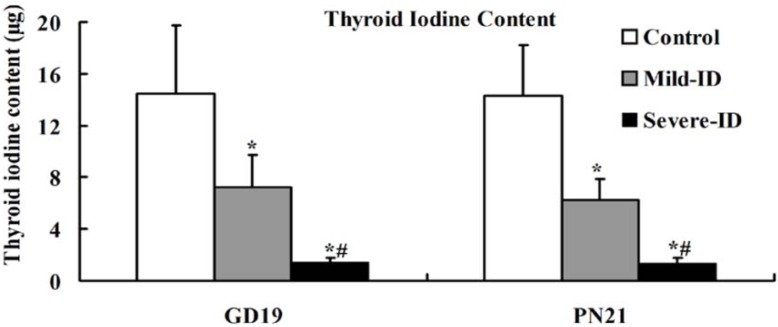
The decreased thyroid iodine content in the thyroid in all groups on GD19 and PN21 (n = 5). * compared to the control, *p* < 0.05; # compared to the mild ID, *p* < 0.05.

To examine whether the mild ID diet can cause mild ID in the thyroid, the thyroid gland weight and thyroid iodine content were measured. Compared with the control group, we observed significantly increased thyroid weights in the mild ID and severe ID groups on GD19 and PN21 (*p* < 0.05, Figure 1). Moreover, thyroid weight in the mild ID group had a significant decrease relative to the severe ID group on GD19 and PN21 (*p* < 0.05, Figure 1). Thyroidal iodine content showed that a significant reduction in the mild ID and severe ID groups relative to the controls on GD19 and PN21 (*p* < 0.05, Figure 2). Thyroid iodine content in the mild ID group was significantly increased relative to the severe ID group on GD19 and PN21 (*p* < 0.05, Figure 2).

### 3.3. Up-Regulated Protein Levels of TTF1 and PAX8 in the Thyroid

**Figure 3:**
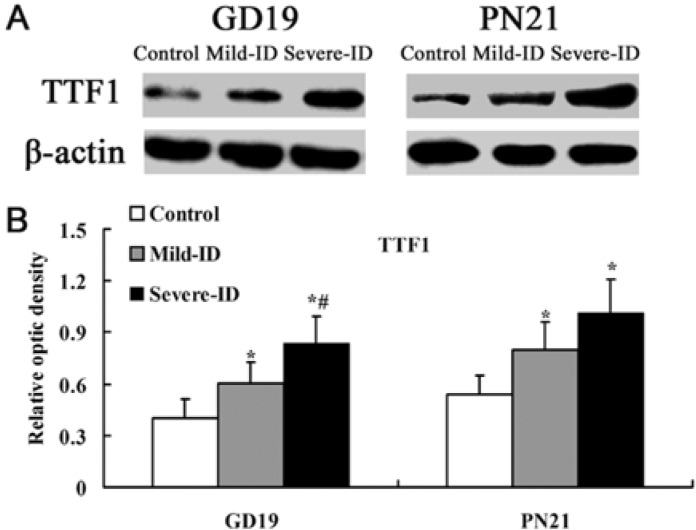
Up-regulated protein levels of TTF1 in the thyroid. The upper bands (**A**) depict representative findings for rats subjected to mild ID and severe ID, respectively. The lower bar graphs show the results of the semi-quantitative measurement of TTF1 (**B**) following mild ID and severe ID treatment. The height of each bar represents the mean ±SD for the groups. At each time point, * compared to the control, *p*< 0.05; # compared to the mild ID, *p* < 0.05 (n=5).

**Figure 4:**
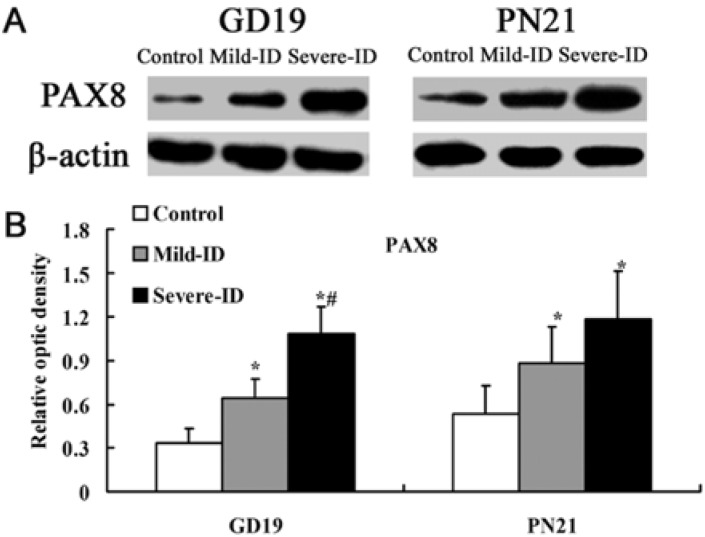
Up-regulatedprotein levels of PAX8 in the thyroid. The upper bands (**A**) depict representative findings for rats subjected to mild ID and severe ID. The lower bar graphs show the results of the semi-quantitative measurement of PAX8 (**B**) following mild ID and severe ID treatment. The height of each bar represents the mean ±SD for the groups. At each time point, * compared to the control, *p* < 0.05; # compared to the mild ID, *p* < 0.05 (n=5).

Transcription factors TTF1 and PAX8 are implicated in thyroid-specific gene transcription [15,24,25]. Compared with the control group, significant up-regulations of thyroidal TTF1 and PAX8 were observed in rats belonging to the mild ID and severe ID groups on GD19 and PN21 (*p* < 0.05, Figure 3(B) and Figure 4(B)).

In addition, the expressions of thyroidal TTF1 and PAX8 in the mild ID group were significantly down-regulated relative to the severe ID group on GD19 (*p* < 0.05, Figure 3(B) and Figure 4(B)).

### 3.4. Up-Regulated Protein Levels of NIS in the Thyroid

**Figure 5:**
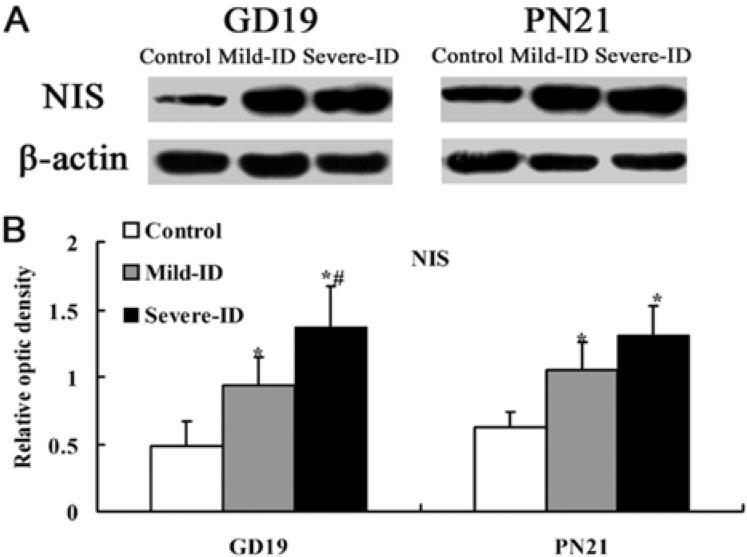
Up-regulated protein levels of NIS in the thyroid. The upper bands (**A**) depict representative findings for rats subjected to mild ID and severe ID. The lower bar graphs show the results of the semi-quantitative measurement of NIS (**B**) following mild ID and severe ID treatment. The height of each bar represents the mean ±SD for the groups. At each time point, * compared to the control, *p* < 0.05; # compared to the mild ID, *p* < 0.05 (n=5).

NIS plays key roles in transporting iodide to the gland [18]. Compared with the control group, the significant up-regulations of thyroidal NIS were observed in rats exposed to the mild ID and severe ID groups on GD19 and PN21 (*p* < 0.05, Figure 5(B)). In addition, the expressions of thyroidal TTF1, PAX8 and NIS in the mild ID group were significantly down-regulated relative to the severe ID group on the GD19 (*p* < 0.05, Figure 5(B)).

### 3.5. Up-Regulated Protein Levels of DIO1 and DIO2 in the Thyroid

**Figure 6:**
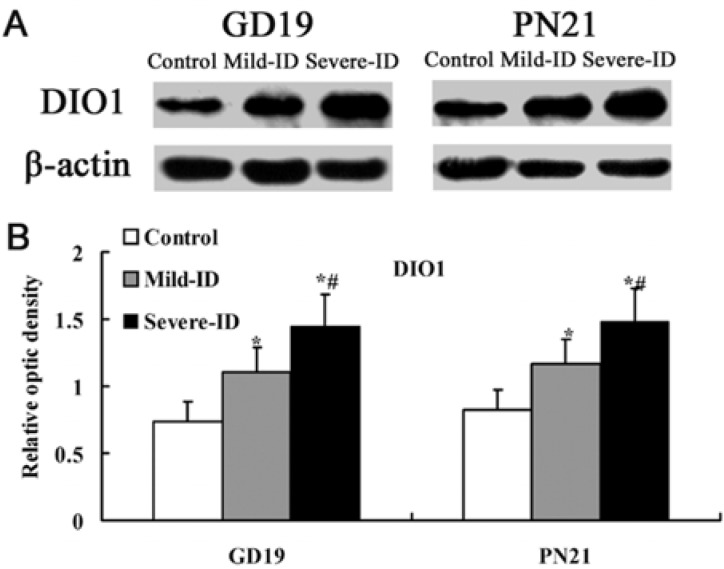
Up-regulated protein levels of DIO1 in the thyroid. The upper bands (**A**) depict representative findings for rats subjected to mild ID and severe ID. The lower bar graphs show the results of the semi-quantitative measurement of DIO1 (**B**) following mild ID and severe ID treatment. The height of each bar represents the mean ±SD for the groups. At each time point, * compared to the control, *p* < 0.05; # compared to the mild ID, *p* < 0.05 (n=5).

**Figure 7:**
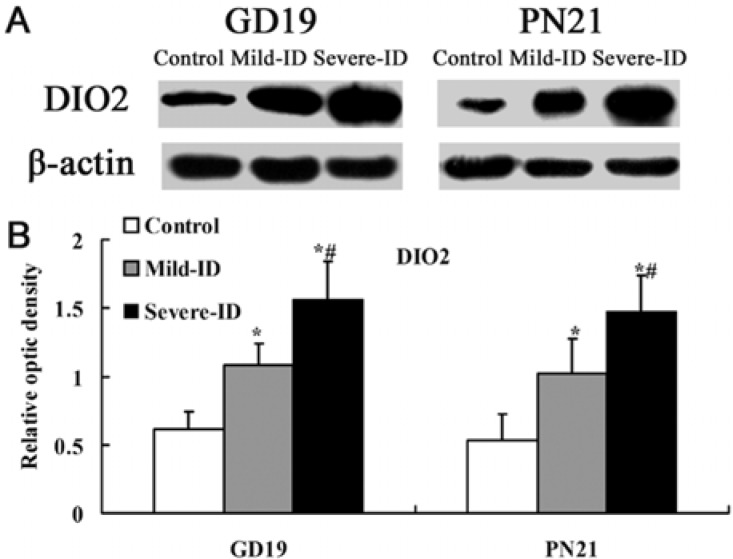
Up-regulated protein levels of DIO2 in the thyroid. The upper bands (**A**) depict representative findings for rats subjected to mild ID and severe ID. The lower bar graphs show the results of the semi-quantitative measurement of DIO2 (**B**) following mild ID and severe ID treatment. The height of each bar represents the mean ±SD for the groups. At each time point, * compared to the control, *p* < 0.05; # compared to the mild ID, *p* < 0.05 (n=5).

DIO1 and DIO2 can regulate TH concentration by determining T3 content in tissues. Compared with the control group, the expressions of thyroidal DIO1 and DIO2 (*p* < 0.05, Figure 6(B) and Figure 7(B)) were significantly increased in rats exposed to the mild ID and severe ID groups on GD19 and PN21. Moreover, the expressions of thyroidal DIO1 and DIO2 in the mild ID group significantly decreased relative to the severe ID group on GD19 and PN21 (*p* < 0.05, Figure 6(B) and Figure 7(B)).

## 4. Discussion

The major findings of this study with that the gestational and lactational dams exposed to mild ID diet were increased thyroid weights, decreased thyroid iodine contents and the up-regulated expressions of thyroidal TTF1, PAX8 and NIS. Moreover, we detected the up-regulation of thyroidal DIO1 and DIO2 in the mild ID group.

ID, the main reason of endemic goiter and hypothyroidism, has historically been a serious public health problem in China [2,26,27]. Due to global iodine screening programs, hypothyroidism induced by severe ID is not very common worldwide, but hypothyroxinemia, a relatively subtle form of TH-deficiency, is still prevalent in developing and developed countries alike [7,8,28]. Maternal hypothyroxinemia may lead to irreversible CNS damage of the posterity in human [9,10]. Therefore, the purpose of present experiment is to explore the effect on thyroidal proteins following mild ID-induced maternal hypothyroxinemia during pregnancy and lactation.

ID causes goiter and several thyroidal changes in rats [29,30]. In the mild ID and severe ID groups, our data showed that the increased thyroid weight and thyroid iodine content declined with the decreasing iodine content in the diet on GD19 and PN21. Therefore, it appeared logical that mild ID diet could induce mild ID in the maternal thyroid during pregnancy and lactation.

To fully understand the impact on thyroid iodine content following mild ID diet, it is necessary to study the expressions of thyroidal TTF1, PAX8 and NIS. In recent years, thyroid transcription factors TTF1 and PAX8 have been shown to be responsible for the expressions of thyroid specific genes, the representative ones being those encoding thyroglobulin, thyroperoxidase and NIS [15,28,31]. It has been demonstrated that TTF1 co-operates with another transcription factor PAX8, which exerts a dominant role in the control of the transcriptional genes in rats and human [32,33]. It has been showed that using the TTF1 antagonist could significantly down-regulate the genes encoding the transcription factors PAX8 in rat thyroid cell line [34]. On the contrary, PAX8 has been reported to control TTF1 gene expression in human thyroid cells [35]. In addition, TTF1 and PAX8-controlled genes expressed in the thyroid gland, as well as the elevated levels of TSH and the thyroidal responsiveness to exogenous TSH [36,37]. NIS could mediate the active iodide uptake to regulate iodide concentration in the mouse thyroid [18]. Endo *et al.* has shown that the promoter activity of rat NIS gene is activated by TTF1 [17]. Moreover, PAX8 has two binding sites of rNIS upstream enhancer and it has an important role in the expression of NIS gene in rat and human [16,38]. PAX8-binding sites also have been described in the promoters of human NIS gene [39,40]. Moreover, it has been showed that NIS activity was increased by TSH in primary cultured human thyroid cells [41]. In line with these literatures, our data clearly showed that mild ID and severe ID groups had the significant up-regulation of thyroidal TTF1, PAX8 and NIS expressions on GD19 and PN21. Moreover, the extent of the increased thyroidal TTF1, PAX8 and NIS levels in mild ID group is less than the severe ID group on GD19 and PN21. It has been demonstrated that re-expression of PAX8 was associated with the recovery of the NIS mRNA expression in a rat thyroid cell line [42]. Furthermore, re-expression of PAX8 could promote iodine accumulation in human tumor cells [35,43]. The PAX8 and TTF1 are necessary to elevate levels of TSH [36]. Because TTF1 and PAX8 synergistically controlled the expression of NIS in rats and human [32,33], we detected both increased thyroid TTF1 and PAX8. Consistent with thyroid iodine content result, we speculated that the synergistically up-regulation of TTF1 and PAX8 increased NIS level to collect iodine for TH synthesis. NIS up-regulation was found in rats following the increased TSH circulating levels induced by propylthiouracil treatment (which inhibits I organification) or an ID diet [1,44]. NIS expression was diminished by hypophysectomy, which exhibited markedly lower TSH levels a rat thyroid cell line [44]. It has been reported that NIS could be increased in the thyroid of fetuses of rats following hypothyroxinemia [45]. Therefore, it is conceivable that the up-regulation TTF1 and PAX8 in our data may increase NIS level and mild ID may up-regulated the levels of TTF1, PAX8 and NIS in the gestational and lactational dams.

In order to further study the effect on thyroidal proteins following mild ID-induced hypothyroxinemia in gestational and lactational dams, we investigated the expressions of thyroidal DIO1 and DIO2. DIO1 and DIO2, the iodothyronine deiodinases, catalyze the deiodination of T_4_ to T_3_ occurring in the phenolic (outer or 5′) -ring of the T_4_ molecule that is a critical step in the TH metabolic process and play a central role in important physiological processes such as maintenance of adequate intracellular T_3_ levels in the different tissues [46]. In the thyroid of non-pregnant female rats following low circulating T_4_ and normal T_3_, it has been shown that thyroidal DIO1 and DIO2 mRNA increased [47]. Since mRNA has to be translated into protein to play functional roles, so further researches on protein levels are needed, especially during pregnancy and lactation. Therefore, in the present study, the significantly increased thyroidal DIO1 and DIO2 levels were detected in the mild ID and severe ID groups on GD19 and PN21. ID leads to a series of physiological adaptations in the hypothalamic-pituitary-thyroid axis, which is an attempt to maintain plasma and tissue T_3_ in the normal range of rat [45]. The up-regulation of rat DIO1 expression contributed to a possible preferential T_3_ secretion [45]. Decreases in peripheral DIO2 might play a role in the fall of serum T_3_ by reducing T_4_ to T_3_ conversion. The rat thyroid preferentially synthesizes and secretes T_3_ according to FT_4_ and FT_3_ thyroidal content [47]. Therefore, on the surface, hypothyroxinemia exposed to the mild ID diet seemed able to maintain normal T_3_ level, but only the decrease of T_4_. However, in order to maintain the levels of the T_3_, thyroidal DIO1 and DIO2 expressions had been up-regulated to promote T_4_ to T_3_ conversion. We speculated that up-regulation of DIO1 and DIO2 expressions were attributed to mild ID and may aggravate hypothyroxinemia in the gestational and lactational dams.

## 5. Conclusions

Our results showed that, in the gestational and lactational dams, the maternal mild ID diet could cause mild ID by impacting thyroidal TTF1, PAX8, NIS. Furthermore, hypothyroxinemia induced by mild ID increased the levels of DIO1 and DIO2.
